# Sentinel lymph node biopsy as guidance for radical trachelectomy in young patients with early stage cervical cancer

**DOI:** 10.1186/1471-2407-11-157

**Published:** 2011-05-02

**Authors:** Xue-lian Du, Xiu-gui Sheng, Tao Jiang, Qing-shui Li, Hao Yu, Chun-xia Pan, Chun-hua Lu, Cong Wang, Qu-qing Song

**Affiliations:** 1 Department of Gynecologic Oncology, Shandong Cancer Hospital, Jinan 250117; Shandong Academy of Medical Science, Jinan 250012; Shandong University, Jinan, 250012; P. R. China

## Abstract

**Background:**

The purpose of this study was to assess the feasibility and accuracy of sentinel lymph nodes (SLNs) detection using 99mTc phytate in predicting pelvic lymph nodes status for radical abdominal trachelectomy (RAT) in patients with early stage cervical cancer.

**Methods:**

Sixty-eight women with stage IA2-IB1 cervical cancer and scheduled to undergo fertility-sparing surgery enrolled in this study. 99mTc-labeled phytate was injected before surgery. Intraoperatively, SLNs were identified, excised, and submitted to fast frozen section. Systematic bilateral pelvic lymphadenectomy and/or para-aortic lymph node dissection was performed. Then RAT was performed in patients with negative SLNs. All nodes were sent for routine pathological examination and immunostained with anti-cytokeratin antibody to detect micrometastases. Outcomes of follow up and fertility were observed.

**Results:**

SLNs were identified in 64 of 68 patients (94.1%). Of these, SLNs of 8 patients (11.8%) were positive on frozen sections and proved to be metastasis by final pathologic examination. The sensitivity, accuracy, and false negative rates were 100%, 100%, and 0%, respectively. All 60 patients with negative SLN underwent RAT successfully. Two relapses occurred and no one died of tumor progression during follow-up. Five of the 15 patients with procreative desire conceived 8 pregnancies (3 term delivery, 2 premature birth, 1 spontaneous abortion, and 2 were still in the duration of pregnancy) after surgery.

**Conclusions:**

The identification of SLN using 99mTc-labeled phytate is accurate and safe to assess pelvic nodes status in patients with early cervical cancer. SLNs biopsy guided RAT is feasible for patients who desire to have fertility preservation.

## Background

Cervical cancer is clinically classified according to the International Federation of Gynaecology and Obstetrics (FIGO) clinical staging system. Although the system does not include evaluation of lymph node involvement, lymph node status is one of the most important prognostic factors in patients with early stage cervical cancer [1-3]. Abdominal radical hysterectomy (RAH) with pelvic lymph node dissection remains the standard of care for early stage cervical cancer [4]. In recent years, radical trachelectomy with pelvic lymphadenectomy is offered to young women with minimally invasive cervical cancer (stage IB1 or lower) with preservation of the uterus in order to maintain fertility [5-7]. This procedure involves a pelvic lymph node dissection and then the surgical resection of the affected cervix and the upper vagina with an adequate margin, and 2/3 of the cardinal and uterosacral ligaments. This is then followed by an endocervical and endometrial sampling above the radical trachelectomy specimen with intraoperative pathologic evaluation of surgical margins.

The sentinel lymph node (SLN) is the first node draining the lymphatic flow from a primary tumor, and represents the status of lymphatic spread [8-10]. Negative SLNs may basically exclude the possibility of the lymph node metastasis of pelvic and other location, and, only on this base, RAT can be considered. Therefore, identification of SLN using 99mTc-labeled phytate can provide a good indication of the feasibility and safety to perform RAT. Women with cervical cancer, particularly younger women who wish to preserve reproductive potential, may be able to avoid radical surgery if sentinel node biopsy is first used to determine the most appropriate treatment option.

The purpose of this study was to assess the feasibility and accuracy of the SLN detection procedure using 99mTc phytate to guide RAT in patients with early stage cervical cancer. We hypothesis that if the SLN concept is valid in cervical cancer, most patients could avoid pelvic lymphadenectomy when the absence of metastasis is intraoperatively confirmed in the SLN. This would definitely benefit many patients by avoiding radical hysterectomy and invasive lymphadenectomy, thus improving life quality and decreasing surgical complications.

## Methods

### Patients and methods

Between March 2003 and December 2009, 68 patients with FIGO stage IA2-IB1 cervical cancer and scheduled to undergo fertility-sparing surgery at the Shandong Cancer Hospital and Institute participated in this study. All patients underwent total pelvic lymphadenectomy. The criteria for preserving the body of the uterus and part of the cervix were as follows: histologic diagnosis of squamous cell carcinoma; fertile age; a desire for future fertility; stage IA2 or IB1; and a cranial extent that would allow for preservation of at least 1 cm of the endocervical canal. The size of the tumor, its location, and cranial extent were assessed in all patients by a combination of magnetic resonance imaging and transrectal ultrasound. The study was approved by the institutional review board, and all patients enrolled in this study provided written informed consent before surgery. Patient characteristics are summarized in Table 1.

**Table 1:** Characteristics of the 68 patients

Characteristics	No. of patients (%)
Mean age (range)	28 yr (18-41)
International Federation of Gynaecology and Obstetrics (FIGO) stage	
IA1	3 (4.4)
IA2	28 (41.2)
IB1	37 (54.4)
≤1.0 cm	12 (32.5)
>1.0 cm, ≤2.0 cm	13 (35.1)
>2.0 cm, ≤3.0 cm	6 (16.2)
>3.0 cm, ≤4.0 cm	6 (16.2)
Cell differentiation	
Well	45 (66.2)
Moderate	14 (20.6)
Poor	9 (13.2)
Childbearing history	
0	38 (55.9)
1	27 (39.7)
≥2	3 (4.4)
Total	68

After the incision is taken down and the specimen is removed, the dilator is kept within the isthmus and a cerclage suture of non-absorbable no. 10 silk suture is performed with 4 or 5 good bites approximately 0.5 cm proximal to the distal end of the isthmus. After surgery, all women were followed up at regular intervals, and combined oral contraceptives were recommended in case of sexual activity. The patients were advised to wait a minimum of 6 months after the procedure, with at least 2 consecutive normal cytology samples, before trying to conceive.

### SLN identification

SLNs were detected with an isotope injection technique into the uterine cervix. On the day before surgery, we injected fluid containing 2.5 ml (100.0 MBq) of ^99m^Technetium-labeled sulfur colloid subepithelially into four quadrants (3, 6, 9, and 12 o'clock positions) of the cervix. The diameter of particle is ranging from 50 to 100 nm. The SLNs were identified intraoperatively by a hand-held gamma detector probe (Neoprobe, neo2000TM, AR-MED, Ltd.) scanning the pelvic side wall, presacral area, and para-aortic lymph node area. Radioactive hot nodes on the basis of counts more than 10-fold above background level were defined as SLNs [11,12]. Then the SLNs were excised with safety margins and submitted to fast frozen section. The radioactivity of the tissue was measured in vivo and after excision, as well as the radioactivity of the surgical bed, to confirm that the marked lesion had been fully excised. After removal of the SLNs, bilateral pelvic lymphadenectomy was routinely done.

RAT was performed if the SLNs were negative. When metastasis was found in the frozen section, hysterectomy and/or para-aortic lymphadenectomy was done to determine the boundaries. Surgical specimens were examined using routine hematoxylin and eosin staining.

### Immunohistochemical staining

All surgically removed lymph nodes including SLNs were immunostained with anti-cytokeratin antibody to detect micrometastases. The intensity of protein expression was evaluated using OPTIMAS 6.5 software.

### Adjuvant chemotherapy

Patients who exhibited one high risk factors according to the pathologic diagnosis received 4 to 6 cycles of adjuvant chemotherapy. The high-risk pathologic features were as follows: poor differentiation, deep stromal invasion, and maximal tumor diameter >2 cm. Adjuvant chemotherapy included either a combination of cisplatin (25 mg/m^2^, d1-3), vincristine (1.25 mg/m^2^, d1, d8), and bleomycin (20 mg/m^2^, d1-3) (combination PVB), or a combination of cisplatin (25 mg/m^2^, d1-3), etoposide (70 mg/m^2^, d1-5), and bleomycin (20 mg/m^2^, d1-3) (combination PEB).

### Follow-up evaluation

Upon treatment completion, patients were evaluated monthly for the first 3 months, every 3 months for the first year, every 6 months during the following two years, and annually thereafter. At each visit, a physical and pelvic examination, blood counts, and chest x-rays were performed. Scans of the abdomen and pelvic region were conducted by Ultrasound (US), computed tomography (CT) scan and/or PET-CT. Survival, recurrence, pregnancy and childbearing information were obtained from personal contact with the patient or her family. Overall survival was calculated from the date of diagnosis. Surviving patients were censored on the date of last follow-up.

### Statistical analysis

Continuous variables were compared with Student's t test, and categorical variables were compared using chi square test or Fisher's exact test, as appropriate. Statistical significance was defined at a level of P < 0.05. All analyses were performed using SPSS version 13.0 (SPSS Inc., Chicago, IL).

## Results

### SLN detection results

The median age was 33 years (ranging from 18 to 41 yrs). A total of 274 lymph nodes were detected as SLNs in 64 of 68 patients, the detection rate was 94.1% (64/68). The number of SLNs identified per patient was 0 in 4 cases, one in 2 cases, two in 6 cases, three in 31 cases, four in 14 cases, five in 11 cases, and six in 8 cases. As shown in table 2, the most common site for SLN detection was the obturator (138 nodes), detected in 45.6% (31/68) patients; followed by the external iliac (86 nodes), detected in 27.9% (19/68) patients; the internal iliac (35 nodes) in 13.2% (9/68); the common iliac (13 nodes) in 5.9% (4/68); and the cardinal ligament (2 nodes) in 1.5% (1/68). No SLN was found in the presacral or para-aortic area. Bilateral pelvic SLNs were detected in 28 of 68 patients (41.2%). The relationships between SLN detection rate and various clinical characteristics are shown in table 3. The SLN detection rate was statistically lower in patients with tumor sizes >3 cm than in patients with tumor sizes ≤3 cm (66.7% vs. 96.8%, *P *= 0.004). Age, FIGO stage, cell differentiation, and preoperative conization did not affect SLN detectability (*P *= 0.153, 0.106, 0.248, and 0.266, respectively).

**Table 2:** The localization and status of the sentinel lymph nodes

SLN detection	64/68 (94.1%)
Total number of SLN	274
Localization of SLN	
Common iliac	13 (4.7%)
External iliac	86 (31.4%)
Internal iliac	35 (12.8%)
Obturator	138 (50.3%)
Cardinal ligament	2 (0.8%)
Sacral	0
Para-aortic	0
Latus of pelvic SLNs	
Unilateral	36/68 (52.9%)
Common iliac	3/36 (8.3%)
External iliac	8/36 (22.2%)
Interanl iliac	4/36 (11.1%)
Obturator	19/36 (52.8%)
Cardinal ligament	2/36 (5.6%)
Bilateral	28/68 (41.2%)
None	4 (5.9%)

SLN, Sentinel lymph node.

**Table 3:** Relationships between SLN detection rate and various clinical characteristics

Characteristic	No. of patients	No. of cases with SLNs detected (%)	SLN detected			*P *value
				
			Unilateral	Bilateral	None	
Age (year)						0.153
≤25	9	9	5	4	0	
>25, ≤30	22	20	12	8	2	
>30, ≤35	20	19	9	10	1	
>35	17	16	10	6	1	
FIGO stage						0.106
IA1	3	3	3	0	0	
IA2	28	26	16	10	2	
IB1	37	35	17	18	2	
Differentiation						0.248
Well	45	44	24	20	1	
Moderate	14	12	8	4	2	
Poor	9	8	4	4	1	
Tumor size						0.004
≤1.0 cm	41	40	22	18	1	
>1.0 cm,≤2.0 cm	13	12	7	5	1	
>2.0 cm,≤3.0 cm	8	8	4	4	0	
>3.0 cm	6	4	3	1	2	
Preoperative conization						0.266
Yes	19	18	11	7	1	
No	49	46	25	21	3	

SLN, sentinel lymph node; FIGO, International Federation of Gynecology and Obstetrics.

**Figure 1:**
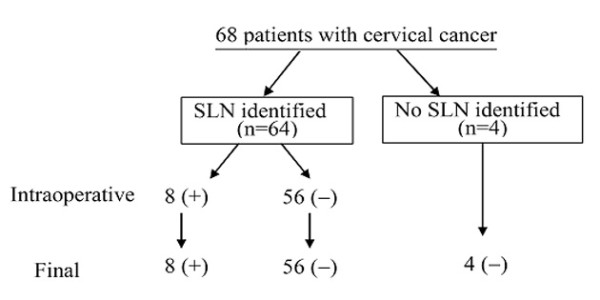
**Outcome of sentinel lymph node biopsy in 68 patients with stage IA2-IB1 cervical cancer**. SLNs were identified in 64 of 68 patients. Of these, SLNs of 8 patients were positive on frozen sections and proved to be metastasis by final pathologic examination. No false negative SLNs were obtained in frozen section.

The results of the frozen sections were identical to those of the permanent staining. As shown in Figure 1, 13 pathologically proven positive nodes were found in 8 (9.3%) patients, which were all SLNs. Four patients had unilateral SLNs and four had bilateral SLNs. Six patients had unilateral lymph node metastasis and two had bilateral metastasis. No false negative SLNs were obtained in frozen section. The sensitivity was 100%, the accuracy and false negative rate was 100% and 0%, respectively, and the negative predictive value of SLN was 100% (Table 4). All of these eight patients with positive lymph nodes were proved to be positive on frozen section. The results of anti-keratinose immunohistochemical staining found no missed micrometastasis in SLNs (data not shown).

**Table 4:** Sentinel lymph node biopsy for evaluation of pelvic lymph node metastasis

SLN	Pathologic diagnosis		
	
	Positive (+)	Negative (-)	Total
Positive (+)	8	0	8
Positive (-)	0	56	56
Total	8	56	64
Results	No. of Patients	% of Total	95% CI
Sensitivity	8/8	100	71-100
Accuracy	64/64	100	89-100
Positive predictive value		100	
Negative predictive value		100	

SLN, sentinel lymph node

### Adjuvant chemotherapy and complications

A total of 88.2% (60/68) patients underwent RAT, whereas 11.8% of patients (8/68) ended up with RAH due to primarily positive SLNs. Patients who underwent RAT and demonstrated high risk factor according to the pathologic diagnosis received 4 cycles (n = 13) or 6 cycles (n = 8) of adjuvant chemotherapy. Of these patients, 12 received chemotherapy of combination PVB and 9 received PEB chemotherapy. For those patients who underwent RAH, 3 received 5 weeks of concurrent chemoradiotherapy and 5 received adjuvant radiotherapy after surgery (data not shown in this paper).

Permanent cerclage during trachelectomy and cerclage at the beginning of pregnancy were placed in 29 and 3 patients, respectively. Surgical complications included post-trachelectomy stenosis of the neo-cervix in 17 of 60 (28.3%) patients, leg lymphedema in 7 of 68 (10.3%) patients, infected pelvic lymphocyst in 5 of 68 (7.3%) patients, and amenorrhea in 3 of 60 (5%) patients.

Side-effects of adjuvant chemotherapy mainly included myelosuppression and gastrointestinal disorders. Grade 3/4 leukopenia occurred in 3 of 21 (14.3%) patients. One (4.8%) patient experienced grade 3 chemotherapy-induced vomiting, nausea and anorexia.

### Outcomes of follow-up

The median time of follow-up was 38 months (range, 3-84 months). Up to March 2010, 7 of 68 (10.3%) patients were lost, leading to follow-up rates amounting to 89.7%. Of the 7 patients lost, 5 were out of contact, another 2 gave up the reexamination. Thus, these patients' responses were not included in the data analysis. The patients lost to follow-up presented no statistically significant differences from the patients who remained in the study in terms of age (P = 0.327), pathologic grade (P = 0.174), tumor size (P = 0.088), preoperative conization (P = 0.169), or complications (P = 0.354, data not shown).

Two relapses occurred at 8 and 17 months after the diagnosis, respectively. One patient occurred with paraaortic lymph node (PALN) metastasis and the other with pelvic recurrence. No one died of tumor progression during follow-up. The actuarial overall survival and disease-free survival at 3 years were 100% and 96.7%, respectively.

Five of the 15 patients who had procreative desire conceived a total of 8 pregnancies within 2 years after surgery. Of the 8 pregnancies, 3 were presented as term delivery, 2 premature births, 1 spontaneous abortion, and 2 were still in the duration of their pregnancies. Two premature births ocurred in 31 and 33 week of pregnancy for premature rupture of membranes (PROM) and the newborns developed well.

## Discussion

In previous studies, SLN detection reportedly had sensitivity ranging from 71% to 100%, a specificity of 100%, and a diagnostic accuracy ranging from 75% to 100% for predicting disease status in the remaining regional lymph node basin [13-16]. In this study, a total of 274 lymph nodes were detected as SLNs in 64 of 68 patients (94.1%). Of these, 8 (11.8%) SLNs were positive and 56 (88.2%) SLNs were negative on intraoperative frozen sections. Final pathologic examination revealed that all 8 patients with positive SLNs had lymph node metastasis. The results of the frozen section were parallel to those of the anti-keratinose immunohistochemical staining. No false negative SLNs were obtained. Our results demonstrated that SLN biopsy was highly sensitive (100%) and accurate (100%) for diagnosing metastases in stage IA2-IB1 cervical cancer. The false negative rate was 0% and the negative predictive value of SLN was 100%. Thus, this study demonstrates that the SLN detection guided RAT is accurate, safe, and efficient to assess pelvic nodes status in patients with early cervical cancer. With careful preoperative evaluation for eligible patients and reliable intraoperative pathological investigation of SLNs, sentinel node navigation surgery can provide large benefits for select patients who desire to have fertility preservation.

Plentl et al [17] described a typical pattern of lymphatic drainage from cervix to regional lymph nodes. According to the pattern, the parametric node is the first draining node in cervical cancer. But the parametric node is too close to the cervix to be detected. Some studies [18,19] reported that 80% of SLNs were located in pelvic structures and the most common site for SLN detection was the external iliac. Our findings were similar to those reports, except that the most SLNs were located in obturator (50.4%, 138/274). Only 31.4% (86/274) SLNs were located in the external iliac. We also detected 35 SLNs in the internal iliac, 13 in common iliac, and 2 in cardinal ligament. No SLNs were found in the parametric region, presacral or para-aortic areas. Unilateral pelvic SLNs were detected in 52.9% of 68 patients in our study. Possible explanations may include that the lymphatic drainage of SLN could occur in one side of pelvis. Similar results were reported by previous studies [20,21].

SLN detection rate was reported to be associated to stage, tumor size, histologic type, preoperative treatment, and a history of preoperative conization. The relationships between SLN detection rate and various clinical characteristics were also investigated in this study. Our results showed that SLN detection was associated with tumor size. SLN detection rate was statistically lower in patients with tumor sizes >3 cm than in patients with tumor sizes ≤3 cm (66.7% vs. 96.8%, *P *= 0.004). A similar trend was reported by Wydra et al. [22].

Different authors hold controversial opinions about whether the preoperative conization has an effect on the detection rate of SLN. Dargent et al. [23] reported that the SLN detection rate, of patients with preoperative conization, was 74%; this was lower than that of patients without preoperative conization (86%). Similar results were reported by Seong et al. [24]. On the contrary, some other authors found that there has been no correlation noted between the preoperative conization and the SLN detection rate [15,25]. Likewise, our data show that detection failure was not influenced by the preoperative conization (*P *= 0.266).

For patients who have high risk factors, such as poor differentiation, deep stromal invasion, or maximal tumor diameter >2 cm, postoperative adjunctive therapy is necessary. Radiotherapy or concurrent chemoradiotherapy are often performed in these patients. However, radiotherapy may destroy the fertility of those patients who underwent RAT, resulting in the failure of fertility preservation. To deal with this problem, we did further studies on the alternative postoperative treatment method for these patients. In one of our previous studies, we compared the efficacy and safety of simple chemotherapy with combined radiotherapy and chemotherapy in 58 patients who exhibited high risk factors after RAH or RAT. Our results revealed that there was no significant difference between the two groups (data not shown). A similar result was obtained in the present study.

Previous studies reported that the recurrence rates of cervical cancer in patients who underwent RAT were comparable to patients who carried out RAH. These recurrence rates ranged from 0% to 8% [25,26]. Similar to these reports, our results demonstrated a low recurrence rate of 3.3% from two patients who relapsed during follow-up (median time of 38 months).

Koliopoulos et al. [25] reported that 210 women who underwent radical trachelectomy had 35 live births after surgery. Shepherd et al. [26] reported that of the 900 cases of women who underwent RAT, over 300 became pregnant resulting in 195 live births. As we know, anticancer drugs injure the female reproductive system through ovarian follicular and stromal damage [27]. With conventional chemotherapy, there is significant differences in ovarian failure rate according to patients age, disease for which patients are treated for, and the drugs used. Some reports discussed the influence of adjuvant chemotherapy on female fertility. Morice et al. [28] reported that the impact of chemotherapy on a woman's fertility depends on her age and the types and doses of the drugs used. Alkylating agents have the biggest negative impact on ovarian function. Protecting fertility potential in females exposed to chemotherapy with GnRH agonists, IVF and embryo cryopreservation or cryopreservation of ovarian tissue is practiced in recent years. Lawrenz et al. [29] reported 1,080 patients received such therapy received 2,417 oocytes and the fertilisation rate per received oocyte was 61.3%. In the present study, five of the 15 patients who had procreative desire were able to conceive after surgery for a total of 8 pregnancies. Premature rupture of the membranes is also a risk, with 10% of babies being born significantly premature. Of those pregnancies in our study, 2 was a premature birth due to the PROM. Cerclage during trachelectomy or at the beginning of pregnancy may help to reduce the rate of miscarriage.

## Conclusions

In conclusion, the current study indicated that, SLN procedure using 99mTc-labeled phytate is a minimally invasive, accurate technique to assess pelvic lymph node status in patients with early cervical cancer. Lymph node mapping using SLN biopsy may help select patients who would benefit from such fertility-sparing surgery. Tumor size is an important factor influencing the success rate of SLN detection. We believe that SLN biopsy guiding RAT is feasible for young patients who desire to have fertility preservation. However, further research is needed to assess the practicality of using these techniques. In addition, long-term survival data for patients with negative sentinel node biopsy are required.

## Competing interests

The authors declare that they have no competing interests.

## Authors' contributions

DXL carried out the clinical studies, participated in the assessment of therapeutic effect and drafted the manuscript. SXG conceived of the study, and participated in its design and coordination and helped to draft the manuscript. JT and LQS carried out the SLN identification. YH and WC performed the follow-up and calculated the overall survival. PCX, LCH and SQQ participated in the design of the study and performed the statistical analysis. All authors read and approved the final manuscript.

## Pre-publication history

The pre-publication history for this paper can be accessed here:

http://www.biomedcentral.com/1471-2407/11/157/prepub

## References

[B1] Benedetti-Panici P Maneschi F Scambia G Lymphatic spread of cervical cancer: an anatomical and pathological study based on 225 radical hysterectomies with systematic pelvic and aortic lymphadenectomy Gynecol Oncol 1996 62 19 24 10.1006/gyno.1996.0184 8690286

[B2] Ho CM Chien TY Huang SH Multivariate analysis of the prognostic factors and outcomes in early cervical cancer patients undergoing radical hysterectomy Gynecol Oncol 2004 93 458 464 10.1016/j.ygyno.2004.01.026 15099962

[B3] Takeda N Sakuragi N Takeda M Multivariate analysis of histopathologic prognostic factors for invasive cervical cancer treated with radical hysterectomy and systematic retroperitoneal lymphadenectomy Acta Obstet Gynecol Scand 2002 81 1144 1151 10.1034/j.1600-0412.2002.811208.x 12519111

[B4] Abu-Rustum NR Hoskins WJ Radical abdominal hysterectomy Surg Clin North Am 2001 81 4 815 828 10.1016/S0039-6109(05)70167-5 11551127

[B5] Hertel H Köhler C Grund D Hillemanns P Possover M Michels W Schneider A German Association of Gynecologic Oncologists (AGO) Radical vaginal trachelectomy (RVT) combined with laparoscopic pelvic lymphadenectomy: prospective multicenter study of 100 patients with early cervical cancer Gynecol Oncol 2006 103 2 506 11 10.1016/j.ygyno.2006.03.040 16690104

[B6] Diaz JP Sonoda Y Leitao MM Zivanovic O Brown CL Chi DS Barakat RR Abu-Rustum NR Oncologic outcome of fertility-sparing radical trachelectomy versus radical hysterectomy for stage IB1 cervical carcinoma Gynecol Oncol 2008 111 2 255 260 10.1016/j.ygyno.2008.07.014 18755500

[B7] Abu-Rustum NR Sonoda Y Black D Levine DA Chi DS Barakat RR Fertility-sparing radical abdominal trachelectomy for cervical carcinoma: technique and review of the literature Gynecol Oncol 2006 103 3 807 813 10.1016/j.ygyno.2006.05.044 16837027

[B8] Morton DL Wen DR Wong JH Economou JS Cagle LA Storm FK Technical details of intraoperative lymphatic mapping for early stage melanoma Arch Surg 1992 127 392 9 1558490 10.1001/archsurg.1992.01420040034005

[B9] Levenback C Coleman RL Burke TW Intraoperative lymphatic mapping and sentinel node identification with blue dye in patients with vulvar cancer Gynecol Oncol 2001 83 2 276 281 10.1006/gyno.2001.6374 11606084

[B10] Levenback C Update on sentinel lymph node biopsy in gynecologic cancers Gynecol Oncol 2008 111 2 Suppl S42 43 18804266 10.1016/j.ygyno.2008.07.029

[B11] Strnad P Robova H Study of lymphatic mapping and sentinel node identification in early stage cervical cancer Gynecol Oncol 2005 98 281 288 10.1016/j.ygyno.2005.04.016 15961145

[B12] Niikura H Okamura C Akahira J Sentinel lymph node detection in early cervical cancer with combination 99mTc phytate and patent blue Gynecol Oncol 2004 94 528 532 10.1016/j.ygyno.2004.05.016 15297199

[B13] Darlin L Persson J Bossmar T Lindahl B Kannisto P Måsbäck A Borgfeldt C The sentinel node concept in early cervical cancer performs well in tumors smaller than 2 cm Gynecol Oncol 2010 117 2 266 9 10.1016/j.ygyno.2010.01.035 20167355

[B14] Yamashita T Katayama H Kato Y Nishiwaki K Hayashi H Miyokawa N Sengoku K Management of pelvic lymph nodes by sentinel node navigation surgery in the treatment of invasive cervical cancer Int J Gynecol Cancer 2009 19 6 1113 1118 10.1111/IGC.0b013e3181a83d65 19820378

[B15] Abu-Rustum NR Neubauer N Sonoda Y Park KJ Gemignani M Alektiar KM Tew W Leitao MM Chi DS Barakat RR Surgical and pathologic outcomes of fertility-sparing radical abdominal trachelectomy for FIGO stage IB1 cervical cancer Gynecol Oncol 2008 111 2 261 264 10.1016/j.ygyno.2008.07.002 18708244 PMC4994885

[B16] Ogawa S Kobayashi H Amada S Yahata H Sonoda K Abe K Baba S Sasaki M Kaku T Wake N Sentinel node detection with (99m)Tc phytate alone is satisfactory for cervical cancer patients undergoing radical hysterectomy and pelvic lymphadenectomy Int J Clin Oncol 2010 15 1 52 58 10.1007/s10147-009-0010-8 20087618

[B17] Plentl AA Friedman EA Lymphatic system of the female genitalia. The morphologic basis of oncologic diagnosis and therapy Major Probl Obstet Gynecol 1971 2 1 223 5162136

[B18] Fader AN Edwards RP Cost M Kanbour-Shakir A Kelley JL Schwartz B Sukumvanich P Comerci J Sumkin J Elishaev E Rohan LC Sentinel lymph node biopsy in early-stage cervical cancer: utility of intraoperative versus postoperative assessment Gynecol Oncol 2008 111 1 13 17 10.1016/j.ygyno.2008.06.009 18684499

[B19] Kara PP Ayhan A Caner B Gültekin M Ugur O Bozkurt MF Usubutun A Sentinel lymph node detection in early stage cervical cancer: a prospective study comparing preoperative lymphoscintigraphy, intraoperative gamma probe, and blue dye Ann Nucl Med 2008 22 6 487 494 10.1007/s12149-008-0144-1 18670855

[B20] O'Boyle JD Coleman RL Berstein SG Intraoperative lymphatic mapping in cervix cancer patients undergoing radical hysterectomy: a pilot study Gynecol Oncol 2000 79 238 243 10.1006/gyno.2000.5930 11063651

[B21] Sheng XG Li DP Liu NF Clinical significance sentient lymph nodes detection in patients with early stage cervical cancer Chin J Obstet Gynecol 2004 39 10 13 14989979

[B22] Wydra D Sawaicki S Wojtylak S Sentinelnode identificationin cervicalcancer patients undergoing transperiton ealradicalhysterectomy: astudyof100cases Int J Gynecol Cancer 2006 16 649 654 10.1111/j.1525-1438.2006.00402.x 16681741

[B23] Dargent D Enria R Laparoscopic assessment of the sentinel lymph nodes in early cervical cancer. Technique--preliminary results and future developments Crit Rev Oncol Hematol 2003 48 3 305 310 10.1016/S1040-8428(03)00129-X 14693343

[B24] Seong SJ Park H Yang KM Kim TJ Lim KT Shim JU Park CT Lee KH Detection of sentinel lymph nodes in patients with early stage cervical cancer J Korean Med Sci 2007 22 1 105 109 10.3346/jkms.2007.22.1.105 17297260 PMC2693543

[B25] Koliopoulos G Sotiriadis A Kyrgiou M Martin-Hirsch P Makrydimas G Paraskevaidis E Conservative surgical methods for FIGO stage IA2 squamous cervical carcinoma and their role in preserving women's fertility Gynecol Oncol 2004 93 469 473 10.1016/j.ygyno.2004.02.002 15099964

[B26] Shepherd JH Milliken DA Conservative surgery for carcinoma of the cervix Clin Oncol(R Coll Radiol) 2008 20 6 395 400 10.1016/j.clon.2008.05.002 18606356

[B27] Meirow D Biederman H Anderson RA Wallace WH Toxicity of chemotherapy and radiation on female reproduction Clin Obstet Gynecol 2010 53 4 727 39 10.1097/GRF.0b013e3181f96b54 21048440

[B28] Morice P Uzan C Gouy S Pautier P Lhomme C Balleyguier C Duvillard P Haie-Meder C Effects of radiotherapy (external and/or internal) and chemotherapy on female fertility Bull Acad Natl Med 2010 194 3 481 92 discussion 492-4, 529-30 21171243

[B29] Lawrenz B Jauckus J Kupka MS Strowitzki T von Wolff M Fertility preservation in >1,000 patients: patient's characteristics, spectrum, efficacy and risks of applied preservation techniques Arch Gynecol Obstet 2010 in press 10.1007/s00404-010-1772-y 21120512

